# Implementing the Bashayer chatbot in Saudi higher education: measuring the influence on students' motivation and learning strategies

**DOI:** 10.3389/fpsyg.2023.1129070

**Published:** 2023-05-15

**Authors:** Ahlam Mohammed Al-Abdullatif, Amany Ahmed Al-Dokhny, Amr Mohammed Drwish

**Affiliations:** ^1^Curriculum and Instruction Department, College of Education, King Faisal University, Al-Ahsa, Saudi Arabia; ^2^Educational Technology Department, College of Specific Education, Ain Shams University, Cairo, Egypt; ^3^Educational Technology Department, College of Education, Helwan University, Cairo, Egypt

**Keywords:** chatbot, artificial intelligence (AI), higher education, motivation, learning strategies, Saudi Arabia

## Abstract

Since the fourth industrial revolution, intelligent software and applications that attempt to mimic human behavior have become increasingly common. The chatbot is an example of an artificial intelligence-based computer program that simulates human behavior by having a conversation and interacting with users using natural language. The implementation of chatbot technology in the educational context is still in its nascent stage, and further investigation into measuring its effectiveness in supporting learning and teaching processes is required, particularly in the context of higher education. Thus, this study presents the design and implementation of a task-oriented chatbot, that is embedded into the WhatsApp application, called Bashayer. It aims at supporting postgraduate students' motivation and learning strategies in Saudi Arabia. A quasi-experimental design with a single-subject experimental approach was adopted with a sample of 60 Saudi postgraduate students. The descriptive analysis of the collected data showed promising results of postgraduate students utilized the Bashayer chatbot system. Participants in the experimental group that used Bashayer were more motivated to learn than those in the control group. Participants also practiced more cognitive and metacognitive learning strategies while utilizing the chatbot compared to the control group. The results of this study are encouraging for the development of chatbot systems similar to Bashayer to support postgraduate students' successful learning. These results contribute to bridging the research gap and adding to the literature on chatbots use in postgraduate educational contexts.

## Introduction

The use of artificial intelligence (AI) technologies in the educational context is increasing. Chatbot technology is an AI application promoted to improve teaching and learning practices (Okonkwo and Ade-Ibijola, 2021). Through conversational interactions and simple user interfaces, chatbots encourage users to actively communicate, explore, and build knowledge (Poncette et al., 2020; Chang et al., 2022). Chatbots are defined as conversational tools that provide users instant services and responses (Smutny and Schreiberova, 2020; Okonkwo and Ade-Ibijola, 2021). According to Pérez et al., “a chatbot is a tool that combines artificial intelligence (AI) and natural language processing or other technology, which enables it to interact to a certain level of conversation with a human interlocutor through text or voice” (2020, p. 1). Chatbots have been gaining popularity in a variety of industries because of their ability to mimic human conversation, which results in automated services and, consequently, reduced human labor (Ondáš et al., 2019; Alhassan et al., 2022; Kuhail et al., 2022b). Joseph Weizenbaum created the first chatbot, ELIZA, in 1966. Since then, the ubiquity of chatbots on the internet has grown rapidly (Fryer et al., 2019). The global chatbot industry is predicted to be worth 1.23 billion dollars by 2025 (Kaczorowska-Spychalska, 2019). Chatbots have the potential to support the field of teaching–learning and act as intelligent tools that accommodate students' habits and needs (Tamayo et al., 2020; Troussas et al., 2022). This is particularly true for higher education contexts, where students are autonomous, self-regulated learners.

Chatbots have the potential to transform the educational landscape by engaging learners, customizing learning experiences, assisting instructors, providing deep insight into learner behavior, and creating a more personalized, engaging learning environment for students (Gonda et al., 2018; Cunningham-Nelson et al., 2019; Bezverhny et al., 2020; Villegas-Ch et al., 2020; Kuhail et al., 2022b). These pedagogical agents allow students to obtain individualized and timely feedback through conversations and be guided through virtual environments (Gonda et al., 2018). Chatbots are becoming increasingly prevalent to promote student learning in e-learning systems (Colace et al., 2018). As support for mobile learning, they are embedded in several e-learning environments, including learning management systems, social network platforms, and digital learning platforms (Wollny et al., 2021; Troussas et al., 2022). Chatbots can instantly provide students with course content, practice questions and answers, assessment criteria, important due dates, academic advising services, directions around campus, and study materials (Mabunda and Ade-Ibijola, 2019; Durall and Kapros, 2020; Okonkwo and Ade-Ibijola, 2021). But these intelligent systems do not stop there; they can also improve student participation and reduce teachers' workloads, allowing the latter to devote more time to curriculum development and evaluation (Cunningham-Nelson et al., 2019).

Several studies have been conducted on the application of chatbot technology to the educational context, such as to answer students' queries, help students learn computer programming concepts, provide assessments of students' performance capabilities, and provide administrative services (Clarizia et al., 2018; Sinha et al., 2020). Moreover, with the rising demand for learning, higher education institutions are under a great deal of pressure to accommodate a larger number of enrolments. As the number of students increases, academic support for students significantly decreases. This has been known to lead to ineffective learning and result in higher dropout rates. Although there are several theoretical solutions to this issue, most are impractical to implement due to budgetary and organizational constraints (Hien et al., 2018). In order to tackle this enormous challenge, tertiary instructors have begun to integrate chatbots into their teaching as pedagogical agents. Chatbots can support individual student learning in large-scale learning environments by assisting the instructor in instantly replying to students' queries, training learners using a variety of learning resources, reintroducing course content and materials, and collecting feedback on training courses (Winkler and Söllner, 2018; Almurtadha, 2019).

Despite the promising results shown by many chatbot application studies in improving the teaching and learning process, their widespread incorporation in higher education contexts is still in its infancy and requires extensive research and study, particularly in the field of how students learn through these smart systems. The current study thus seeks to investigate the effect of a task-oriented chatbot system called Bashayer, which is embedded into WhatsApp, in supporting postgraduate students' motivation and learning strategies.

## Significance and contribution of the study

The COVID-19 pandemic has had a drastic effect in changing the higher educational landscape. Among the most prominent effects is greater reliance on mobile technology and its applications for learning, particularly with the growing number of mobile users and increasing internet availability around the globe (Bahja et al., 2020; Almurayh, 2021). This has led to a significant increase in the use of AI-based tools in education, especially chatbots as a conversational system that serve remote learning (Troussas et al., 2019). For example, Sandu and Gide (2019) confirmed that in the 2021 academic year, 48.9% of higher education students in India used chatbots to communicate with their academic institutions. The study predicted that chatbots will become the most popular technology to solve students' educational problems due to their increased availability and convenience. According to Hien et al. (2018), chatbots can assist higher education institutions in enhancing their existing services, minimizing staff expenses, and providing new and innovative services. Chatbot technology implementation in teaching–learning practices is anticipated to be ubiquitous in the future. This is particularly true for higher education and is driven by the current emphasis on digital transformations in the direction of AI-enhanced learning environments imposed by the fourth industrial revolution and fifth-generation technologies. In this context, this research study is motivated by the recommendations of existing literature, which has identified the need to integrate smart AI-chatbot systems into the education sector to implement student-centered learning and address various learning challenges (Sandu and Gide, 2019; Heryandi, 2020; Sjöström and Dahlin, 2020). In their recent systematic literature review, Kuhail et al. (2022b) recommended that future investigations should focus on exploring the impact of chatbots on learner satisfaction and learning effectiveness.

Despite the large body of research that measured the effects of chatbot technology on teaching and learning practices, most studies have been conducted on undergraduate and K-12 students. There is a scarcity of chatbot research with postgraduate students that measures its impact on their motivation and learning strategies. Due to the nature of postgraduate education, and the emphasis on students being self-learners, self-motivators, and self-regulators of their learning strategies, it is important to measure chatbot technology's effect on learning motivation and strategies at this level. Furthermore, a thorough investigation of related literature reveals that motivation and learning strategies have not been comprehensively explored in the postgraduate context. Most chatbot studies focus on measuring impact on general learning motivation and cognitive skill acquisition. Hence, our study is unique in its use of the framework developed by Pintrich (1991) to comprehensively measure the Bashayer chatbot system's influence on motivational orientation through task value, self-efficacy, and cognitive and metacognitive learning strategies. Additionally, there has been a paucity of empirical research conducted in Saudi higher education environments. Only two studies, conducted by Alqaidi et al. (2021) and Al-Ghadhban and Al-Twairesh (2020), were found to explore the effects of chatbots in supporting Saudi undergraduate students' learning enquires. However, these studies did not focus on measuring actual learning practices or students' motivational orientations toward chatbots. Therefore, the results of our study will contribute to bridging the research gap in the field of chatbot applications in the Saudi higher education context.

To achieve this goal, the current study aims to integrate the Bashayer chatbot system into a social learning platform via WhatsApp and investigate its effect on the motivation and learning strategies of postgraduate students at King Faisal University, Saudi Arabia. It intends to contribute to the literature on integrating chatbot technologies into education by focusing on two significant factors: learning motivation (task value and self-efficacy for learning and performance) and learning strategies (cognitive and metacognitive self-regulation). These results will contribute to bridging the research gap and adding to the pool of existing literature on chatbot use among postgraduate students. These findings will shed light on the potential of such intelligent tools in increasing learners' motivation and facilitating the practice of learning strategies both cognitively and metacognitively.

## Literature review

### The chatbot technology and its architecture

Chatbots rely on a variety of frameworks that govern their operations. For example, chatbots developed by Microsoft differ from those developed by Facebook (Manaswi, 2018). Although they both have the same purpose of receiving instant messages, they differ in the programming languages used in their development, the type of conversations they provide to the user, and the data models stored in their databases (Hwang and Chang, 2021). Chatbot architecture can be divided into two types. The first is task-oriented chatbots. These are concerned with accomplishing a specific task, and they are prepared in a way that answers the user's inquiries through dialogue. The second is non-task oriented chatbots, which aim to add humor and fun to ordinary conversations without a commercial purpose (Hussain et al., 2019; Vijayakumar et al., 2019).

Hwang and Chang (2021) identified two key components of the chatbots that engage with users using natural language. First, the user message analysis component determines the user's intentions and purpose. Second, the response generation component provides the user with an appropriate response depending on the context and information available to it from the existing dialogue (e.g., user data, geographical area, login times, and clicks to navigate within the chatbot system). In this manner, appropriate responses are presented to the user considering the intent of the conversation and contextual information (Hwang and Chang, 2021). Studies have shown that chatbots have three types of appropriate response generation models: pattern-based, retrieval-based, and generative models (Manaswi, 2018). The pattern-based model generates appropriate responses depending on the exact match between the question and the answer stored in the database. The retrieval-based model has a higher degree of flexibility and relies on available queries and analyses, such as the context of the user information and data. The generative model relies on analyzing past and current user contexts to generate appropriate responses.

In the current study, the proposed Bashayer chatbot system employs the retrieval model to provide increased flexibility and support in the educational services it provides to postgraduate students. It is also a task-oriented chatbot built to attain a set of learning goals through interactions with postgraduate students in their courses.

### The adoption of chatbot technology in education

Chatbots integration into e-learning environments over the past decade reflects an increasing interest in their utility in teaching–learning modalities (Troussas et al., 2019; Smutny and Schreiberova, 2020). Pedagogically, chatbots can be used as conversers, helplines, and recommendation tools (Lin and Chang, 2020). According to Pérez-Marín (2021), chatbots may engage with students in the role of advisors, tutors, classmates, or gamers. These innovative tools can promote student learning motivation, cognitive skill acquisition, and overall performance (Lin and Mubarok, 2021; Okonkwo and Ade-Ibijola, 2021; Pérez-Marín, 2021; Fidan and Gencel, 2022). Learners can develop their skills by using chatbots to assess their behavior and keep track of their advancement (Colace et al., 2018). Chatbots' consistency, availability, and accessibility as conversational agents facilitate active interactions, which result in engaging experiences for learners (Sriwisathiyakun and Dhamanitayakul, 2022). Such easy and flexible interactions, powered by chatbot technology, enhance autonomous learning, learning engagement, goal orientation, learning strategies, and achievement (Winkler and Söllner, 2018; Durall and Kapros, 2020; Pérez et al., 2020; Smutny and Schreiberova, 2020; Du et al., 2021; Haristiani and Rifai, 2021). In addition, chatbots have the potential to activate students' problem-solving and critical thinking skills (Goda et al., 2014; Pérez-Marín, 2021; Cabrera et al., 2022), develop learning self-efficacy and self-organization (Durall and Kapros, 2020; Pérez et al., 2020), promote stress management and self-direction (Park et al., 2019), and enhance self-regulation in education (Calle et al., 2021; Cabrera et al., 2022).

Chatbot technology has many positive effects and great potential for learning and teaching applications. The chatbot-based environment greatly accentuates how content and materials are presented through the segmentation of learning modules and the organization of learning tasks. Thus, it supports autonomous learning, which is when a learner is able to determine their own learning priorities (Pérez et al., 2020; Haristiani and Rifai, 2021). This allows them to decide what, how, and when to learn, thus supporting the principles of mastery learning (Troussas et al., 2019). This can be facilitated by offering various activities and enabling students to engage step by step in learning while providing continuous support and feedback, which ultimately allows learners to master the required knowledge and skills. This makes chatbots' learning environments full of educational opportunities characterized by quality and efficiency. Chatbots also promote participatory learning and the exchange of learning materials between individuals regardless of temporal and special boundaries. In addition, they provide immediate support for learning activities by peers within the same learning environment. This results in the enhancement of the concept of personalized learning by providing learning modules that are compatible with students' cognitive styles (Okonkwo and Ade-Ibijola, 2021; Troussas et al., 2022). Furthermore, chatbots support mobile learning and thus benefit from constant availability. They are thus considered to be a practical application of the concept of ubiquitous learning (Heryandi, 2020; Sjöström and Dahlin, 2020). Chatbot applications are characterized by a familiar user interface that relies on the dialogue style, which is a human characteristic, and easy operating systems that students use in their daily life, such as Android or Apple. Chatbots depend on the transfer of knowledge to the learner through dividing and displaying content in a way that facilitates memorization, retrieval, and, thus, mastery of learning. Chatbots also rely on innovative methods to provide tests, assessments, and feedback that are compatible with mobile devices' physical characteristics (Troussas et al., 2020; Wollny et al., 2021).

Many researchers have designed and developed chatbots for various educational contexts and using different sample populations, but they all have one common goal: to evaluate the influence of these intelligent systems on the effectiveness and quality of teaching and learning practices. Findings from recent empirical research on chatbot use to support teaching and learning has been summarized in Table 1.

**Table 1:** Recent empirical studies on integrating chatbot technology to promote teaching and learning.

**Study**	**Purpose**	**Sample and context**	**Key findings**
Troussas et al. (2019)	Exploring the effect of a developed chatbot in supporting personalized learning experience	K-12/language learning/mobile learning	Promising results in supporting students' learning process.
Pereira et al. (2019)	Investigating the influence of using a chatbot to promote learning motivation and peer-to-peer assessment	Undergraduate students/massive open online course (MOOC)/ higher education	The use of a chatbot improved peer assessment and overall learning. Ninety percent of the students support using chatbots in their future classes.
Nghi et al. (2019)	Assessing the effectiveness of using chatbots to support learning engagement and performance	Undergraduate students/language learning/higher education	The majority of students indicated the potential for chatbots to enhance their learning process. They also indicated that learning became more fun and exciting with chatbots.
Chen et al. (2020)	Examining the influence of employing a designed chatbot for acquiring Chinese language.	Undergraduate students /language learning (Items)/higher education	Students confirmed that the chatbot supported their Chinese item acquisition. Significant improvement in students' learning achievements.
Lin and Chang (2020)	Exploring the effect of chatbots on promoting writing skills	Post-secondary writers/language learning (writing)/k-12 education	Chatbot has a positive influence on students' writing skills and peer feedback process.
Mendoza et al. (2022)	Evaluating a proposed chatbot system to support teacher-student interaction.	Third-year middle school students and teachers/online learning/k-12 education	Third-year teachers and students indicated positive outcomes of using a chatbot to support student-teacher interaction.
Lee et al. (2020)	Investigating the use of a developed chatbot as an online instructor in a computer science course	Undergraduate students/online learning higher education	Students showed a positive reaction toward the use of a chatbot as an online tutor and suggested its integration into other e-learning platforms.
Chuah and Kabilan (2021)	Examining the perceptions of English teachers toward using chatbot technology to support teaching-learning process *via* a mobile learning environment.	English teachers/mobile learning/teacher education	Chatbot was useful in providing feedback, stimulating interaction cycles, and increasing the level of social presence that results in an active learning environment.
Deveci Topal et al. (2021)	Investigating the influence of chatbots on attitudes and learning achievement.	Fifth-grade students in science course/k-12 education	Chatbot has no significant effect on learning achievement. Students indicated the usefulness and enjoyment of using chatbots, which resulted in a positive online learning experience.
Kumar (2021)	Exploring the effectiveness of using educational chatbots on learning performance within a collaborative learning environment.	Undergraduate students/online learning higher education	Educational chatbots improved learning performance, collaboration, and teamwork. However, there was no impact on self-efficacy, motivation, or cognition.
Yin et al. (2021)	Investigating the impact of a chatbot within a micro-learning environment on motivation level and learning performance.	Undergraduate students enrolled in micro-learning/higher education	Chatbot increased students' intrinsic motivation and learning performance.
Chang et al. (2022)	Assessing the influence of a mobile chatbot to improve learning performance and self-efficacy among nursing students	Nursing students/professional training program	Chatbot improved nursing students' learning performance, engagement, success, and self-efficacy.
Fidan and Gencel (2022)	Exploring the impact of a chatbot embedded in instructional videos to support learning performance and enhance intrinsic motivation	Undergraduate pre-service teachers/online learning/higher education	Chatbot enhanced preservice teachers' intrinsic motivation and learning performance.
Khalil and Rambech (2022)	Investigating students' perception toward an integrated chatbot in Telegram learning-based platform	Undergraduate students/online learning/higher education	Students found learning with the chatbot was easy, useful, efficient, and engaging.
Kohnke (2022)	Exploring the influence of chatbot use in supporting distance learning	Undergraduate students/distance learning/higher education	Students considered the instructional chatbot helpful in giving a sense of human interactions; reduced feelings of loneliness resulted in better learning engagement.

Most of these studies have been conducted in the context of higher education and employ undergraduate students as the sample population. Few have been developed to assess the effect of chatbots on students' learning in K-12 education. These studies have been carried out in different educational contexts and learning environments, including distance learning (Kohnke, 2022), online learning (Lee et al., 2020; Troussas et al., 2020; Mendoza et al., 2022), mobile learning (Troussas et al., 2019; Chuah and Kabilan, 2021), massive open online courses (MOOCs) (Pereira et al., 2019), micro-learning (Yin et al., 2021), teacher education (Chuah and Kabilan, 2021), professional training (Chang et al., 2022), K-12 language learning (Chen et al., 2020; Lin and Chang, 2020; Troussas et al., 2022), and science education (Deveci Topal et al., 2021). The vast majority of chatbots have been employed as teaching agents and assessed using the experimental approach, and the findings largely point to improved learning performance and satisfaction (e.g., Pereira et al., 2019; Chen et al., 2020; Yin et al., 2021; Khalil and Rambech, 2022). Only two studies reported chatbot use to have a non-significant effect on learning outcomes (Deveci Topal et al., 2021), cognition, motivation, and self-efficacy (Kumar, 2021).

From a teaching perspective, many studies have indicated that using chatbots on teaching practices yields positive results. For example, Gonda et al. (2018) discussed the use of a chatbot developed to advance the teaching process, particularly learning assessment. Wu et al. (2020) found that chatbots within e-learning platforms outperformed teachers in acting as a helpline service. Huang et al. (2021) evaluated the use of chatbots pedagogical and social contexts. They discovered that chatbots appeared to promote students' social presence through open, emotive, and coherent dialogue. Bii et al. (2018) investigated teachers' attitudes toward employing chatbot technology for teaching–learning purposes, and the results were found to be highly positive. Hobert (2019) designed Coding Tutor, a tutoring chatbot system, to enhance the teaching of a programming course. It was found to be successful in assisting both teaching and learning. In general, in most studies, a positive trend was observed toward an increase in the use of chatbots in future education (Pereira et al., 2019; Lee et al., 2020; Chuah and Kabilan, 2021; Fidan and Gencel, 2022; Khalil and Rambech, 2022; Mendoza et al., 2022).

### Related works

Song et al. (2017) designed and developed a chatbot system to encourage meaningful interactions between graduate students in online courses. Their findings demonstrated that immediate interactions related to course content between learners and chatbot systems are best suited to graduate-level online courses. Alkhoori et al. (2020) developed the UniBud chatbot, which provided students with voice interaction systems for academic advising. UniBud was found to support a restricted range of academic queries, which permitted academic advisors to answer more involved questions. Kuhail et al. (2022a) developed MyAdvisor, a chatbot for academic advising that used real-world advising scenarios. Students were found to quickly understand it and found it useful. Calle et al. (2021) developed a chatbot-based recommendation system to help students self-manage their learning by delivering suggestions for time, sessions, resources, and activities inside a virtual platform to achieve better outcomes. All four studies dealt with measuring chatbots systems' capabilities in terms of their efficiency in supporting interactions with and academic advising for university students. Given the importance of measuring chatbots' influence on actual learning processes, the current study differs from this literature pool. It empirically measures chatbots' effectiveness in developing motivation levels and supporting cognitive and metacognitive learning strategies.

Another study by Abbasi and Kazi (2014) found that chatbot use had a positive effect on students' memory retention and learning performance. Goda et al. (2014) demonstrated that chatbot use improved students' critical thinking skills and learning engagement in learning the English language. Bailey et al. (2021) created Storybot, a narrative-focused chatbot for language acquisition, and it was found to enhance the students' reading comprehension. They indicated that Storybot was easy to navigate and helped them achieve their learning goals. The current study agrees with this body of research in focusing on the impact of chatbot systems on the learning process, such as achievement and learning engagement. However, it varies from these studies in that it comprehensively measures motivation level through task value and self-efficacy as well as cognitive and metacognitive learning strategies using the conceptual framework by Pintrich (1991). Also, the current study employs a sample of postgraduate students, unlike previous studies that focused only on undergraduate students.

Chatbots are widely utilized in the context of language learning because they provide a free and accessible form of language interaction (Fryer et al., 2019; Haristiani, 2019). Troussas et al. (2017) developed the ALICE chatbot to teach English while providing integrated support to students' learning and assessment. The results indicated that the built-in conversation was beneficial to the mobile learning experience, and the students' feedback was very encouraging. This study is similar to the current research in the possibility of providing students continuous learning support and educational content in a variety of forms. The current study is also characterized by its provision of multiple types of immediate or deferred support and feedback. Haristiani and Rifai (2021) developed Gengobot, a chatbot-based Japanese grammar learning system that was demonstrated to be an engaging, creative, and interactive tool for autonomous learning. Lin and Chang (2020) developed a chatbot to promote the writing skills of post-secondary writers, and they concluded that it has positive effects. They discovered that chatting with chatbots during lessons motivates students to learn, and chatbots make learning easier, more manageable, and more enjoyable. All the aforementioned studies developed chatbot systems and tested their impact on the language learning outcomes of K-12 students. The current study has the advantage of examining chatbot system effectiveness in a different context of language learning with postgraduate students.

Troussas et al. (2020) developed a smart educational application through Facebook called i-LearnC#. It aimed to support student learning by relying on the virtual trainer method to establish a private teaching platform. The application employed the cluster analysis method to recommend optimal groups for student cooperation. The results indicated that the application was educationally beneficial for higher-education students, and it worked to promote effective learning through its role as an adaptive, smart, social learning environment. This study is similar to our current study in that our study relies on the WhatsApp application, and the virtual tutor's method. The current study differs in that it employs the Bashayer system in the context of postgraduate studies and focuses on measuring cognitive and metacognitive learning strategies instead of only determining their impact on enhancing learning and the consequent extent of acceptance.

Troussas et al. (2022) presented an educational application based on mobile learning to provide interactive educational activities and motivational feedback for primary school students to develop their cognitive skills. The results revealed the efficiency of the designed application in developing students' cognitive skills and internal motivations. This study aligns with our current study in its focus on measuring motivation and cognitive learning, but the current study deals with a different study population of postgraduate students. Furthermore, it focuses not only on the acquisition of cognitive skills but also on chatbots' impact in supporting both cognitive and metacognitive learning skills.

## Methodology

The current study followed a quantitative approach, which examines an educational phenomenon from the participants' point of view (Leedy and Ormrod, 2005). Therefore, a quasi-experimental design was adopted to determine the relationship between the current study's variables, which mainly depends on the field rather than laboratory experimentation (Neuman and Robson, 2014). The study relied on single-subject experimental methods based on studying the effect of the independent variable on the dependent variables (Neuman and McCormick, 1995). It aimed to investigate the effect of the Bashayer chatbot system (independent variable) integrated into a social network (WhatsApp) to support both motivation and learning strategies (dependent variables). Therefore, the current study attempts to answer the following research questions: (1) Does the use of the Bashayer chatbot system enhance postgraduate students' learning motivations? (2) Does the use of the Bashayer chatbot system enhance postgraduate students' learning strategies?

**Figure 1:**
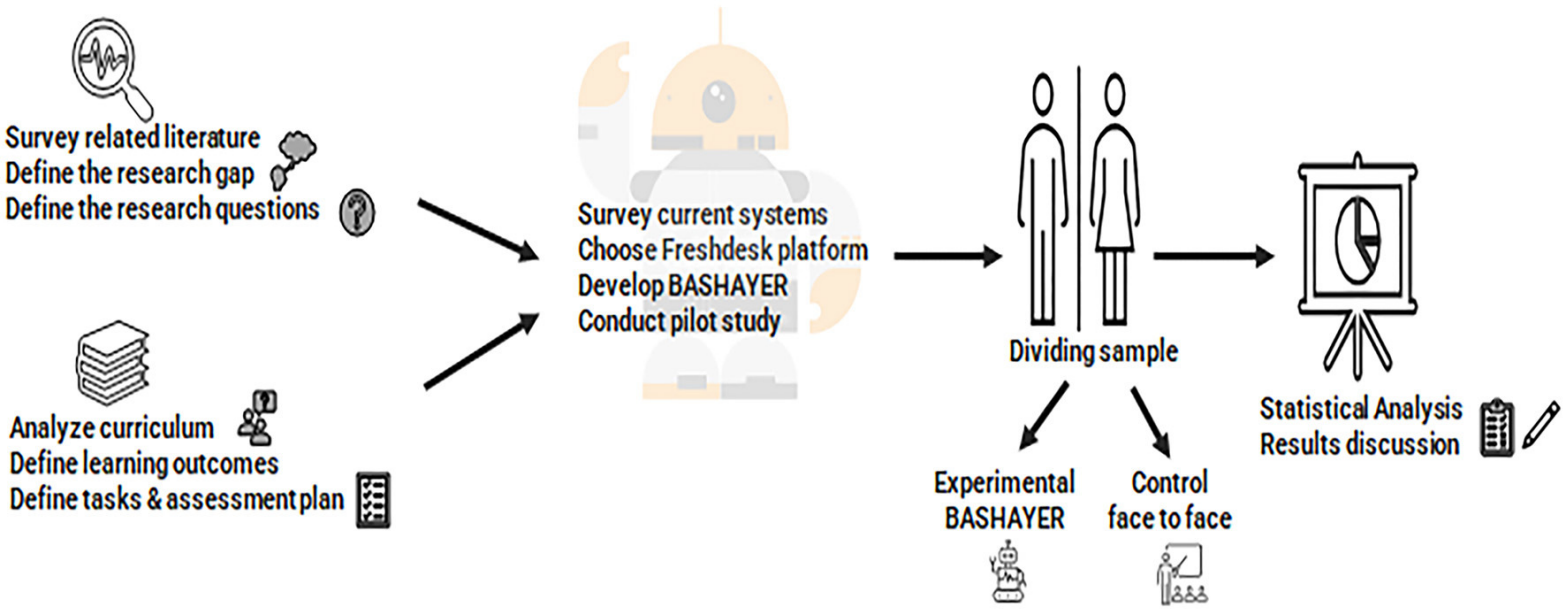
Research methodology.

This study's procedures are outlined in Figure 1. It began by analyzing the relevant literature and identifying the research gap and research questions. Following this, the targeted courses were analyzed in terms of defining learning outcomes, learning tasks, and assessment methods. Current chatbot systems were then reviewed and a platform (Freshdesk) was determined accordingly. The Bashayer chatbot system was designed and exported on the platform. A pilot study was conducted to evaluate the initial design and propose any changes and required amendments. Next, the student sample was divided into two study groups, the control and the experimental, and Bashayer was applied to the experimental group. Finally, the study survey instrument was administered to the two groups, and the necessary statistical measurements were calculated to answer the research questions.

### Setting and sample

The study sample consisted of male and female Saudi postgraduate students enrolled in the Master of Educational Technologies program at the College of Education at King Faisal University. Participants were enrolled in two courses: Reading in Educational Technologies in English Language and Dissertation. A purposeful random sample of 70 male (*N* = 15) and female (*N* = 55) students was selected. The participants' ages ranged from 21 to 30 years, and all were enrolled in the first semester of the 2022–2023 academic year. For the purpose of this study, the sample was divided into 10 postgraduate students in the pilot study, 30 postgraduate students in the experimental group (who were taught via online mode using the Bashayer chatbot system), and 30 postgraduate students in the control group (who were taught via traditional face-to-face mode). The participants had to provide informed consent to participate in this study and submit responses to the questionnaires, which ensured their voluntary participation, privacy, and data confidentiality. All their information was used for scientific research purposes only.

### Measurement

To achieve the purpose of this study, the motivated strategies for learning questionnaire (MSLQ) was utilized (Pintrich, 1991). It is a self-reported instrument developed to investigate learning motivation and learning strategies. The survey questionnaire consists of two main scales with a total of 38 items: the motivation scale (14 items) and the learning strategies scale (24 items). The motivation scale includes two subscales: task value (6 items) and self-efficacy for learning and performance (8 items). According to Pintrich (1991), task value refers to the degree that students perceive the course material to be interesting, important, and useful, whereas self-efficacy refers to students' confidence and ability to successfully perform learning tasks. The learning strategies scale (24 items) includes two subscales: cognitive learning strategies (12 items) and metacognitive self-regulation learning strategies (12 items). Cognitive learning strategies in this context refers to the cognitive processes that students perform when learning. It ranges from simple practices, such as remembering (listing and naming) and understanding (paraphrasing and summarizing), to the practice of organizing (clustering and outlining), to critical thinking (reasoning and problem solving). However, for the purpose of this study, we focus on cognitive learning strategies that include remembering, understanding, and organizing, while the metacognitive self-regulation learning strategies relate to control and self-regulation. These entail students planning, monitoring, and regulating their own learning. Examples include setting goals, tracking cognitive learning, self-testing, and checking and correcting learning as they progress through a task. The participants were asked to respond to the 38-item MSLQ using a 5-point Likert scale. The scale score ranged from 38 (lowest) to 190 (highest).

The MSLQ survey questionnaire has been empirically validated by Pintrich (1991). For this study, the questionnaire was translated into Arabic; therefore, the validity of its content was assessed. This was ascertained by consulting the opinion of three experts who specialized in educational technology and were proficient in English. They examined the translation in terms of meaning accuracy and consistency with the original MLSQ items. The experts deemed the majority of the 38 items to be accurate and advised a few items be rephrased for improved clarity. After following these suggestions, the instrument was finalized.

The statistical reliability of the MSLQ was measured by calculating each subscale's internal consistency using Cronbach's alpha coefficients on a pilot sample of 10 postgraduate students who were randomly selected and excluded from the study sample. The values of Cronbach's alpha for all subscales ranged from 0.81 to 0.86, which indicates a good level of reliability (Field, 2013). Furthermore, the reliability was further checked by administering the MSLQ instrument on the pilot sample two distinct times. After 2 weeks had passed since the first administration, the scale was again applied to the same pilot sample to calculate Pearson's correlation coefficient. The correlation value between the two applications was 0.84, which represents high reliability. Table 2 shows the alpha coefficients for the pilot study sample and current study sample and compares them to the original scale by Pintrich (1991). In the current study sample, the Cronbach's alpha was 0.85 for the motivation scale and 0.87 for the learning strategy scale, which demonstrated a high degree of reliability for this study's instrument.

**Table 2:** MLSQ scale reliability test.

**MLSQ**		**Item No**.	**Pilot study α**	**Current study α**	**Original scale α**
**Scale**	**Subscale**				
Motivation scale	Task value	6	0.78	0.80	0.75
	Self-efficacy for learning and performance	8	0.84	0.86	0.70
	Total	14	0.81	0.85	0.73
Learning strategies scale	Cognitive	12	0.81	0.82	0.72
	Metacognitive self-regulation	12	0.85	0.87	0.79
	Total	24	0.83	0.86	0.76

### Data collection

To conduct the current study, approval was obtained from the Scientific Research Ethics Committee at King Faisal University under the number KFU-REC-2022-SEP-ETHICS184. To implement the Bashayer chatbot in two master's degree courses, the researchers developed an integrated teaching plan and review for each course. Then, the weekly lesson plans were disseminated at the beginning of semester one of the academic year 2022–2023. An integrated assessment plan was also developed, including weekly assignments and final projects. To assess the homogeneity between the two sample groups, at the beginning of the course, all participants (*n* = 60) completed the MLSQ survey questionnaire as a pre-test. Once the application of the Bashayer chatbot system with the experimental group ended at week 13, its participants completed the post-test.

### Experimental procedures

**Figure 2:**
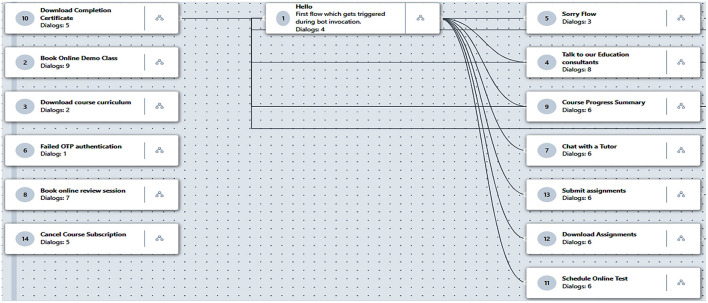
Navigation flow map for the Bashayer chatbot system.

In order to assess the design and content validity of the proposed Bashayer chatbot system prior to the experiment, an initial version was presented to three experts who were selected for their extensive experience with information and communication technologies. The purpose of the chatbot, the nature of the courses, and the intended learning outcomes were presented and discussed. The navigation flow map of the Bashayer chatbot system (shown in Figure 2) was also explained to the experts, who then expressed their opinions about the chatbot's appropriateness for the study purpose and target group. They suggested amendments to certain design elements, such as backgrounds, font sizes, and student registration system, which they believed would enhance readability. Furthermore, to support validity, five postgraduate students within the pilot sample were asked to navigate the system to provide suggestions for improvement. The students suggested some modifications to the location of the main menu buttons, and they were adjusted accordingly. After the authors implemented the recommendations, the final version of the Bashayer chatbot was created.

**Figure 3:**
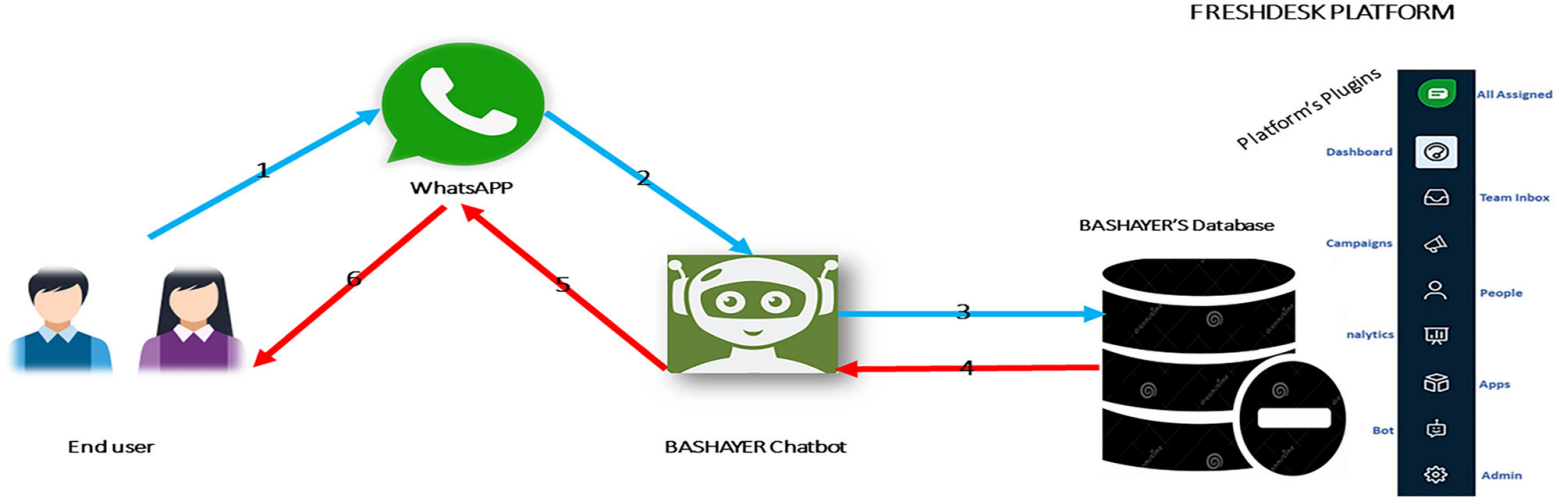
How the Bashayer chatbot system works.

The Bashayer chatbot system was implemented in the study according to Figure 3. The students of the experimental group interacted with the system through WhatsApp using text conversations. The chatbot was produced and hosted using the Freshdesk platform, which enables the free integration of chatbots into social media applications. WhatsApp was chosen due to its immense popularity as a text messaging application; it is even the most popular among university students. After specifying the name of the chatbot, it was linked to the teacher's WhatsApp number and then made available to participants. The teacher began communicating with the students through his own dashboard on the Freshdesk platform. Thus, Bashayer was managed by the teacher using Freshdesk as a host platform. After the students interacted with Bashayer through WhatsApp, their questions and inquiries about the course were directed to Freshdesk, which the system was hosted on. The answers were then searched for in the system database, which was also attached to the platform. Finally, the appropriate responses were directed to the students through Bashayer via WhatsApp.

To initiate the experiment, postgraduate students of the experimental group were introduced to the Bashayer chatbot system and informed about its purpose, its use, and how to communicate with it through WhatsApp. The 11 functions included in the chatbot were explained to the participants, the first of which was the welcome and electronic registration “hello” function. Through this, postgraduate students could log in to Bashayer. This information was stored in the system database separately for each student in order to perform subsequent functions on it, such as registration and course enrolment. If such information has not been recorded, the system asks the user to record it. The second function was the electronic registration for the course, the “book online” demo class function. This function enables postgraduate students to electronically register for courses via the Bashayer chatbot system and provides instructors with a list of those registered with them. The third was the “download curriculum” function, where postgraduate students were given the opportunity to download course plans, materials, and lectures.

The fourth function was the “talk to our education consultant” function. Through this, the postgraduate students were given the opportunity to talk with their teachers and obtain answers to their repeated, periodic, or detailed inquiries and questions about the course, whether at the level of the course content and materials or in terms of coordination. To receive responses from the teacher, the chatbot system asked postgraduate students to select their preferred means of communication, such as their university email or by phone via their WhatsApp registered number. The fifth function was “book online review session.” Postgraduate students were given the opportunity to book an electronic review session to discuss their weekly duties and provide feedback on the assignments provided to them. This could be done individually, and sometimes, more than one student would gather within the same session. Sixth was the “course progress summary” function. The students were given the opportunity to download all the course progress summaries, worksheets, and references on a weekly basis. Seventh was the “download assignment” function. This function allowed students to download weekly assignments through the chatbot by clicking on external links that were renewed weekly through the system. All weekly assignments were made first to the control group via the learning management system (Blackboard). Then, all assignments were provided to students in the experimental group as external links to the Blackboard, due to the limited download space provided by the Freshdesk free version that was used for the experimental group. When students clicked the assignment's external link, it downloaded directly without displaying the Blackboard environment. Therefore, students interacted only with the chatbot learning environment. Students then uploaded their responses to the chatbot, which was stored on the Freshdesk platform. Managing the process of learning for the experimental group was done in isolation from the Blackboard environment.

The eighth function was the “submit assignment” function. Postgraduate students could upload their assignments for the teachers to review and evaluate through Freshdesk. This made it possible for instructors to display the list of students who completed their weekly duties and the means of communication by which they chose to receive feedback. Ninth was the “schedule online test” function. The students were given the opportunity to choose appropriate dates and times to hold midterm exams from several available days through Bashayer. Tenth was the “download completion certificate” function. This allowed the students to download the course-completion certificate after taking the final exam. Eleventh was the “cancel course subscription” function. The postgraduate students were given the opportunity to cancel their course registration within the first 2 weeks of the first term.

Finally, if any data was entered by the students, and there was no appropriate response registered in the system database for it, the system issued the message, “Sorry, I can't fulfill your request now.” The student was then either transferred to the main menu to start again or a ticket was left for the course instructor. The instructors could log in to the Freshdesk platform at their own convenience to respond to the inquiries, as all student data was available on the platform.

## Findings

**Figure 4:**
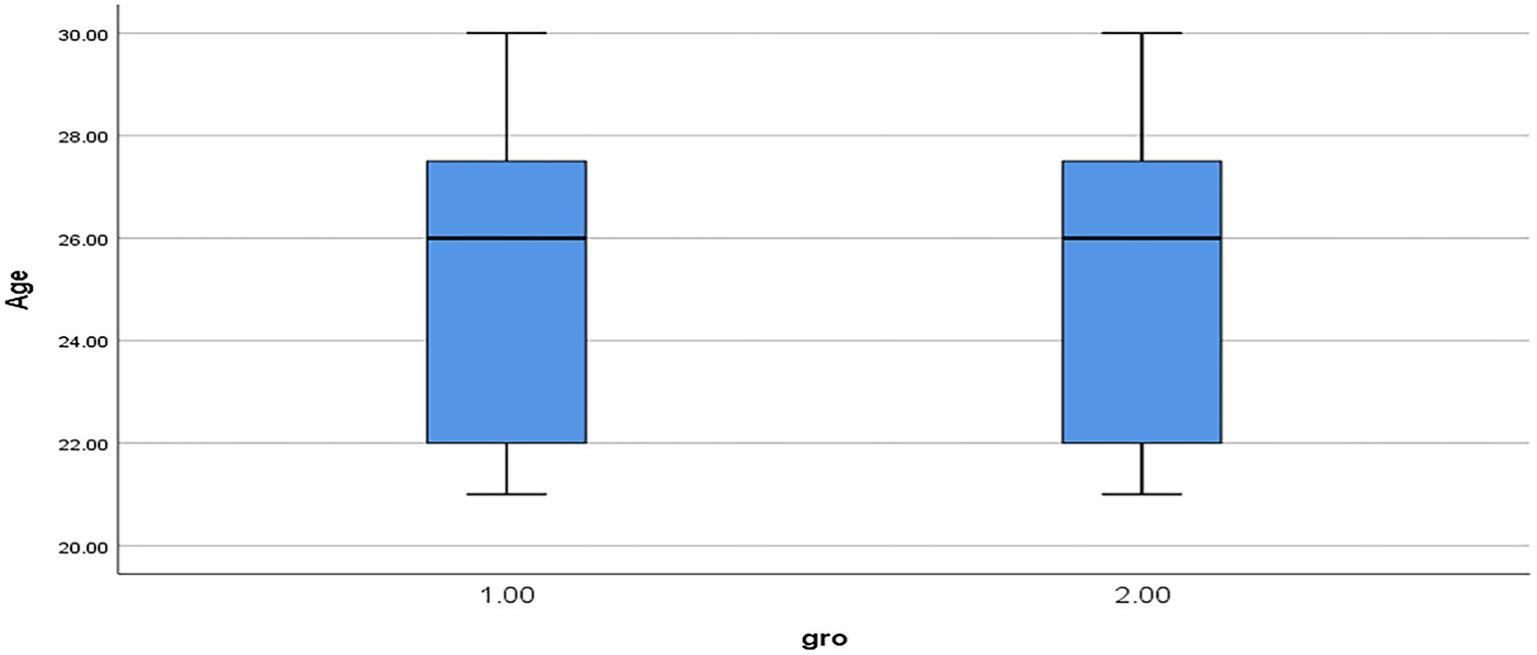
The statistical mean distribution for the study sample in terms of age.

To measure the homogeneity of the study sample, we calculated the independent samples *t*-test on both groups' pre-test responses. No statistically significant difference (*t* = 0.097, *p* > 0.05) was found between the control group (M = 12.83, SD = 2.704) and experimental group (M = 12.76, SD = 2.608). This confirms the homogeneity of the study sample. Furthermore, in terms of the age variable, Figure 4 shows the mean and standard deviation of the control group (M = 24.97; SD = 0.49) and the experimental group (M = 24.82; SD = 0.48), which indicates that the statistical distribution for the study sample is normal. The Kolmogorov-Smirnov test score was 0.20, the Shapiro-Wilk test score was 0.15, and *p* > 0.05. Therefore, the two groups were homogeneous before the experiment began and follow normal distribution of data.

To carry out the statistical analysis of the current study, data collected from the post-test, which was conducted after the experiment, were tabulated and entered in IBM SPSS v.26 software. The statistical analysis followed two steps. First the descriptive statistics (M, SD, and SE) of the two MSLQ scales (motivation and learning strategies) and their four subscales (task value, self-efficacy for learning and performance, cognitive learning strategies, and metacognitive self-regulated learning strategies) were calculated. Second, the independent samples *t*-test were performed to measure the statistical differences between the mean scores of the two groups. The two research questions and their associated findings are discussed below.

**Figure 5:**
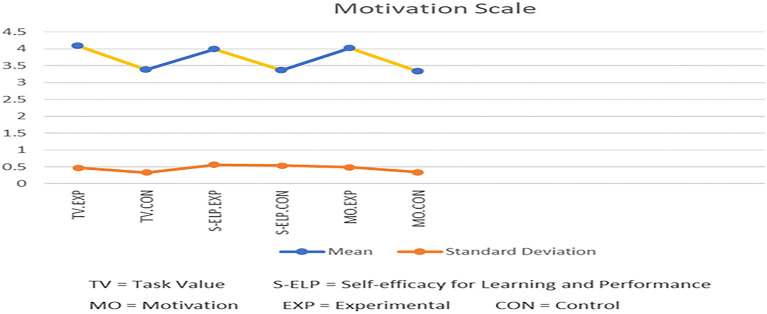
Descriptive statistics for the two groups in MSLQ motivation scale (*N* = 60).

The first research question is as follows: does the use of the Bashayer chatbot system enhance graduate students' learning motivation? Table 3 and Figure 5 show a comparison of the descriptive statistics (M, SD, and SE) for postgraduate students' responses in both the control and experimental groups according to the two MSLQ motivation subscales of task value and self-efficacy for learning and performance. As shown in Table 3, postgraduate students' mean response for task value was 0.7 greater in the experimental group (4.08) than the control group (3.38). Similarly, in terms of self-efficacy for learning and performance, the mean response of the postgraduate students from the experimental group (3.99) was 0.6 greater than those from the control group (3.37). In terms of the MSLQ motivation scale in total, the students from the experimental group (M = 4.03) showed 0.7 greater level of motivation than their peers in the control group (M = 3.34).

**Table 3:** Comparison between the two groups in MSLQ motivation scale (*N* = 60).

	**Control group**			**Experimental group**			***T*** **-test**		
**Subscale**	***M***	***SD***	***SE***	***M***	***SD***	***SE***	***df***	***t***	***sig***
Task value	3.38	0.33	0.06	4.08	0.47	0.08	58	6.64	0.000
Self-efficacy of learning and performance	3.37	0.54	0.09	3.99	0.56	0.10	58	4.33	0.000
Total for motivation scale	3.34	0.34	0.06	4.03	0.49	0.09	58	6.25	0.000

The result from the independent samples *t*-test, as shown in Table 3, indicates that there was a statistically significant difference between the two groups in terms of postgraduate students' responses for the task value (*t* = 6.64, *p* < 0.05) and self-efficacy for learning and performance (*t* = 4.33, *p* < 0.05) questions of the MSLQ motivation scale (*t* = 6.25, *p* < 0.05), which favored the experimental group. This means that postgraduate participants in the experimental group (who used the Bashayer chatbot system) were more motivated to accomplish their learning tasks than those in the control group. These results confirm the positive effect that using the Bashayer chatbot has on postgraduate students' learning motivations.

**Figure 6:**
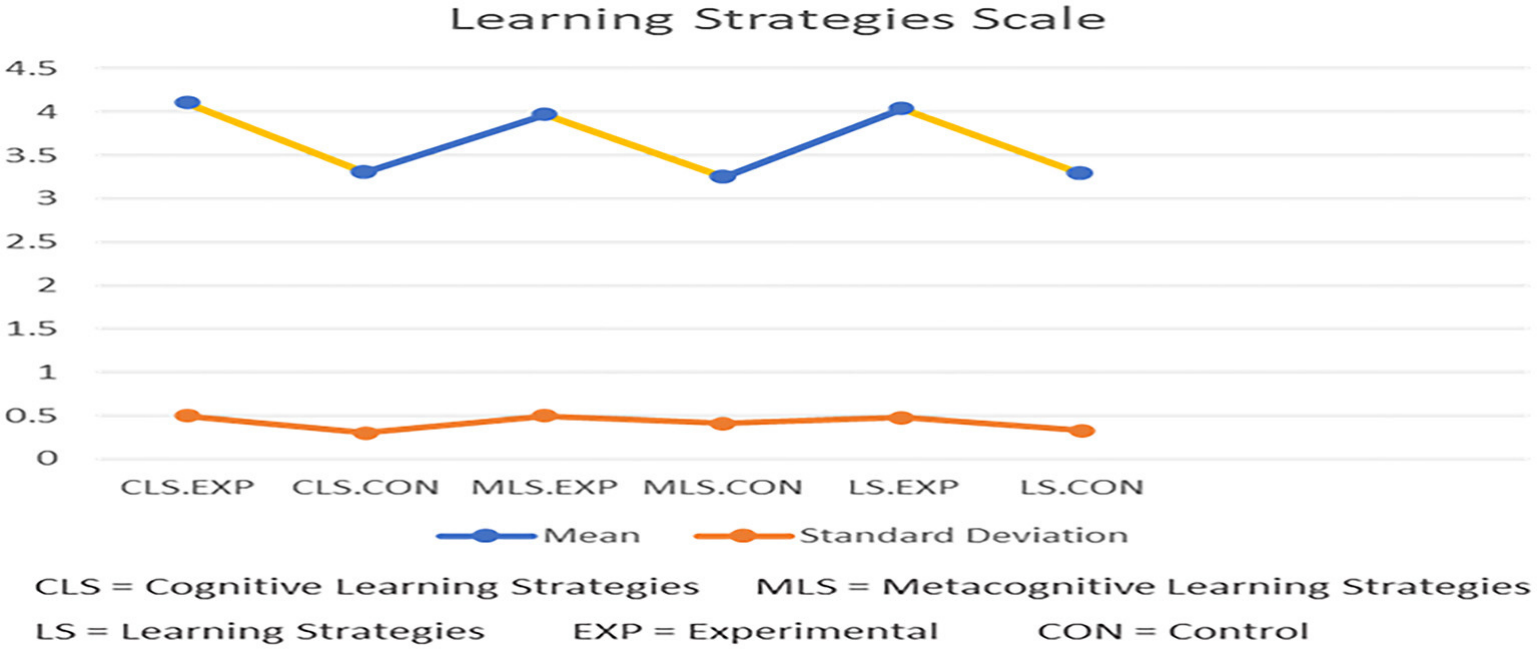
Descriptive statistics of the two groups for the MSLQ learning strategies scale (*N* = 60).

The second research question is as follows: does the use of the Bashayer chatbot system enhance graduate students' learning motivation? Table 4 and Figure 6 show a comparison of the descriptive statistics (M, SD, and SE) of students' responses in both the control and experimental groups according to the two MSLQ learning strategy subscales: cognitive and metacognitive self-regulation learning strategies. In terms of cognitive learning strategies, the mean response of the postgraduate students from the experimental group (4.11) was 0.8 greater than the control group (3.33), as shown in Table 4. Similarly, in terms of metacognitive self-regulation learning strategies, the mean response of the postgraduate students from the experimental group (3.97) was 0.7 greater than those of the control group (3.25). In terms of the total MSLQ learning strategies scale, the mean response of the postgraduate students from the experimental group (4.04) was 0.8 higher than the control group (3.29).

**Table 4:** Comparison between the two groups in MSLQ learning strategies scale (*N* = 60).

	**Control group**			**Experimental group**			***T*** **-test**		
**Subscale**	***M***	***SD***	***SE***	***M***	***SD***	***SE***	***df***	***t***	***sig***
Cognitive learning strategies	3.33	0.30	0.06	4.11	0.51	0.09	58	7.18	0.000
Metacognitive learning strategies	3.25	0.41	0.08	3.97	0.51	0.09	58	6.07	0.000
Total of learning strategies scale	3.29	0.33	0.06	4.04	0.48	0.09	58	6.89	0.000

Furthermore, the result from the independent samples *t*-test, as shown in Table 4, indicates a statistically significant difference between the two groups in terms of their responses to the practices of cognitive learning strategies (*t* = 7.18, *p* < 0.05) and metacognitive self-regulation learning strategies (*t* = 6.07, *p* < 0.05) of the MSLQ learning strategies scale (*t* = 6.89, *p* < 0.05). This favored the experimental group. This means that postgraduate participants using the Bashayer chatbot system and its functionalities were able to better cognitively practice learning and metacognitively regulate their learning strategies. This confirmed that Bashayer chatbot use positively influenced postgraduate participants' learning strategies.

## Discussion

The focus of this study was on understanding the effect of a developed Bashayer chatbot system on learning motivation and learning strategies among postgraduate students in Saudi higher education. The study findings are discussed in the following section, and its several implications are suggested accordingly.

The results of the study indicated that postgraduate students in the experimental group demonstrated higher levels of motivational orientation toward learning when using the Bashayer chatbot system than their peers in the control group. Postgraduate students' motivational orientation was assessed using two elements: task value and self-efficacy for learning and performance. In terms of task value, postgraduate students viewed their learning experience using the Bashayer chatbot system as being more motivating, meaningful, convenient, and beneficial as compared to the traditional learning environment. These results align with previous research studies on chatbot systems' potential to support learning motivation (Nghi et al., 2019; Chen et al., 2020; Yin et al., 2021; Fidan and Gencel, 2022; Khalil and Rambech, 2022). In terms of self-efficacy for learning and performance, the results indicated that the chatbot enhanced students' confidence and ability to successfully perform and accomplish learning tasks compared to in a conventional learning environment. Similar results have been found in previous studies such as Chen et al. (2020), Pérez et al. (2020), and Chang et al. (2022).

The positive effect of the Bashayer chatbot on postgraduate students' learning motivation may be attributed to its design and embedded features, which create an accessible, flexible, and convenient learning environment (Sjöström and Dahlin, 2020). For example, the chatbot provided a consolidated presentation of relevant content, materials, resources, and assignments for students to view learning procedures, details of all learning tasks, and due dates. It also allowed students to balance between handing over their assignments and devoting themselves to subsequent learning steps and procedures. Instant and continuous feedback via the “inquiry ticket” feature was among those that helped raise students' motivation. The postgraduate students initiated questions that were answered from the lecturer's interface. Responding to students' questions and inquiries greatly helped in guiding them and increasing their involvement in learning activities. Providing feedback and stimulating the interaction cycle that supports social presence are important factors in active engagement with the chatbot learning environment (Lee et al., 2020; Huang et al., 2021; Kohnke, 2022; Mendoza et al., 2022). Moreover, the Bashayer chatbot system provided postgraduate students with a weekly summary of what had been taught over the past week, which the students could review and use to conveniently follow up on learning activities. This helped to bridge students' learning gaps in subjects that had not yet been mastered, thus building confidence in Bashayer's potential to support learning performance. The students were thus motivated by this learning environment that they considered important and of great benefit, which was as advocated by the learning-based chatbot technology of many studies (Pereira et al., 2019; Yueh and Chiang, 2020; Kumar, 2021; Chang et al., 2022; Troussas et al., 2022).

The results of our study indicated that postgraduate students who used the Bashayer chatbot demonstrated a better level of practicing learning strategies than their control group peers. Postgraduate students' learning strategies were evaluated using two elements: cognitive learning strategies and metacognitive self-regulation learning strategies. In terms of the former, the students using the Bashayer chatbot system were able to practice various strategies, even more than those who studied in the traditional environment. This signifies that cognitive learning strategies, such as remembering (listing and naming), understanding (paraphrasing and summarizing), and organizing (clustering and outlining) were enhanced by the learning environment created by the Bashayer chatbot. Despite recent studies (e.g., Deveci Topal et al., 2021; Kumar, 2021) that found no significant influence of chatbot use on K-12 and undergraduate students' cognition and learning achievements, the results of our study highlight the significant potential of chatbot-based learning environments in enhancing and supporting cognitive learning strategies among postgraduate students. This positive result could be attributed to the instant, flexible retrieval of course information (Chuah and Kabilan, 2021), which might positively influence students' memory retention (Abbasi and Kazi, 2014), skill acquisition, and overall learning performance (Lin and Mubarok, 2021; Okonkwo and Ade-Ibijola, 2021; Pérez-Marín, 2021).

Similar results were found in terms of metacognitive self-regulated learning strategies. The results showed that the Bashayer chatbot system enhanced postgraduate students' confidence and ability to be more self-regulated and exert more control over their learning strategies when compared to their peers in the traditional learning environment. Through the chatbot learning environment, postgraduate students were able to better plan, manage, and monitor their learning, including setting goals, tracking cognitive learning, self-testing, and self-checking. These results confirm other studies' findings about chatbot systems' potential to support metacognitive learning strategies, such as self-regulation (Calle et al., 2021; Du et al., 2021; Cabrera et al., 2022), self-organization (Pérez et al., 2020), and self-direction (Park et al., 2019).

Through the Bashayer chatbot system, several features were made available for students to control and manage their own learning. For example, it provided a reference to the learning record feature for each student, which is a record of students' information saved in the application's database. This contains everything related to the students' data on their learning process, such as their assignments, submission dates, grades, feedback, and previous inquiries. This gave students the confidence that their learning steps were recorded and saved so that they could be followed up by the teacher anytime and anywhere. The layout and navigation of the Bashayer chatbot system contributed to reducing distractions and cognitive load. This facilitated the practicing of cognitive learning strategies, such as repeatedly viewing and memorizing course readings; extracting information from resources; and understanding, summarizing, and organizing them at their own pace. In terms of metacognitive self-regulation learning strategies, the postgraduate students were able to track and monitor their own learning progress to adjust and correct their behavior as they learned.

## Implications, limitations and future work

The present study and its findings have yielded several important implications. The results imply that chatbot system designers should focus on providing students with customized, personalized learning environments (Cunningham-Nelson et al., 2019; Bezverhny et al., 2020; Krouska et al., 2020; Yueh and Chiang, 2020). The more meaningful the learning environment is for students, the more motivated to learn, and the more involved in learning activities, they will be (Fryer et al., 2019; Yin et al., 2021). This can be achieved through the inclusion of more intelligent functions that can analyse student-entered data and their interaction styles within the chatbot to better support learning. In addition, the results of this research confirm that when designing a chatbot system, its construction should reflect and be properly aligned with conceptual course maps, learning outcomes, and students' characteristics. Also, it should be designed to provide levels of curiosity, challenge, and mastery. Therefore, chatbots should be designed according to students' cognitive learning styles (Sjöström and Dahlin, 2020). All the learning activities carried out by the students should be targeted to achieve the best outcomes, thus building students' confidence in chatbot-based learning environments.

There are several limitations in the current study. First, due to the quasi-experimental nature of this study, the small sample of 60 postgraduate students limits the possibility of generalizing the obtained results to the wider population. Future studies should thus conduct descriptive studies with a larger sample size of the target population. Second, in terms of context, the nature of the courses that the Bashayer chatbot system was implemented in is theoretical. Therefore, the results may vary when the chatbot is applied to courses from other disciplines that are of an applied or practical nature. Accordingly, it is advisable to design and test a chatbot system specifically for such courses and measure its effectiveness on postgraduate students' motivation and learning strategies. In addition, the experiment was conducted only over a short duration of one semester. Our courses are taught in a one-semester system and not extended throughout the year. Therefore, we propose conducting future research when the application period is extended across one or two academic years and includes a variety of courses. This should reduce the potential negative impacts of novelty and eliminate potential biases due to a group's exceptionality in one semester. Third, this study relied on a quantitative methodology through the application of the MSLQ scale; therefore, judgments are based on quantitative data only. Qualitative data would have provided a deeper, richer understanding of the phenomenon. This is especially true for the subject of learning strategies and whether students practice them through cognitive or metacognitive self-regulation, as well as regarding chatbots as an artificial intelligence tool that simulates human performance. Therefore, tools such as observation, interviews, and reflective notes would have helped gain a greater understanding of postgraduate students' experiences with the Bashayer chatbot system. It is recommended that mixed methods research studies be conducted in the future to better understand chatbot technology adoption in learning contexts.

Fourth, there were limitations in terms of the architecture and functionalities of the Bashayer chatbot system. In the current study, the database that fed Bashayer with necessary data was not always enough to answer all students' queries. Therefore, it is recommended that future chatbot system designs be based on more sophisticated algorithms that enable the designer to expand the range of data stored within them and, thus, generate varied responses commensurate with students' questions. Fifth, the current system relied on databases. These were used to provide a specific set of responses in line with the nature of the content and tasks provided through the application, which could be somewhat limiting. Therefore, the current study recommends including interactive databases or a set of libraries based on activating a recommendation system to provide responses. On the one hand, these can be related to discussions and human vocabulary, and on the other hand, they can help provide creative solutions related to the various learning procedures within the chatbot environment.

Furthermore, it is suggested that future research should focus on measuring more complex cognitive learning strategies, such as reasoning and problem-solving. The Bashayer chatbot design was limited to the basic cognitive skills of remembering, understanding, and organizing, with control and self-regulation as the metacognitive skills. Additionally, communication between students and their instructors was conducted through traditional means such as email, phone, or texting via WhatsApp. To further enhance the role of the teacher within the chatbot learning environment, future designs can include an avatar for the instructor (in the form of a virtual teacher) that simulates the instructor's personality and provides support and feedback to students. Furthermore, for future designs, the chatbot systems can include functions that analyse students' facial features, personal expressions, and communication skills and generate appropriate responses to provide a more personal learning experience. Moreover, these future design could incorporate smart strategies to classify students according to either their cognitive styles or previous experiences that are necessary for new learning.

## Conclusion

The adoption of AI-based tools, such as chatbots, to support teaching and learning has proliferated. This study's main goal was to determine the influence of a chatbot system named Bashayer on postgraduate students' learning motivation and learning strategies. A quasi-experimental design was implemented with a sample of 60 Saudi postgraduate students divided into experimental and control groups. The MSLQ survey questionnaire (Pintrich, 1991) was utilized as the main instrument for this study. Learning motivation was assessed by measuring postgraduate students' perceptions toward two main subscales: the task value (the interest, usefulness, and importance of course material) and self-efficacy for learning and performance (the confidence and ability to perform learning tasks). The results showed that the students in the experimental group (who were using the Bashayer chatbot system) revealed a higher level of perceived task value and self-efficacy for learning and performance than those in the control group. Likewise, learning strategies were instigated by measuring postgraduate students' perceptions toward two main subscales: cognitive learning strategies (remembering, understanding, organizing, and reasoning) and metacognitive self-regulation learning strategies (control and self-regulation of learning). The results indicated that by using the chatbot system, participants in the experimental group demonstrated more favorable levels of both cognitive and metacognitive learning strategies than their peers in the control group. All results obtained in this study demonstrated the positive influence of the Bashayer chatbot system, an AI-based tool, on enhancing motivation and learning strategies for Saudi postgraduate students. These results are promising in terms of the potential of chatbot adoption to enhance learning motivation and support cognitive and metacognitive learning strategies among postgraduate students. This study offers motivating results that support the development of more chatbot systems that are similar to Bashayer to support successful learning.

## Data availability statement

The raw data supporting the conclusions of this article will be made available by the authors, without undue reservation.

## Ethics statement

The studies involving human participants were reviewed and approved by the Scientific Research Ethics Committee at King Faisal University under the ethics number (KFU-REC-2022-SEP-ETHICS184). The patients/participants provided their written informed consent to participate in this study.

## Author contributions

AAl-A, AAl-D, and AD contributed to conception and design of the study and implemented the experimental procedures and collected data. AAl-A obtained the ethics and funding of the study and wrote the first draft of the manuscript. AAl-D and AD performed the statistical analysis and wrote sections of the manuscript. All authors contributed to manuscript revision, read, and approved the submitted version.
